# Geographical Variability in Mortality in Urban Areas: A Joint Analysis of 16 Causes of Death

**DOI:** 10.3390/ijerph18115664

**Published:** 2021-05-25

**Authors:** Miguel A. Martinez-Beneito, Carlos Vergara-Hernández, Paloma Botella-Rocamora, Francisca Corpas-Burgos, Jordi Pérez-Panadés, Óscar Zurriaga, Elena Aldasoro, Carme Borrell, Elena Cabeza, Lluís Cirera, Josu Delfrade Osinaga, Ana Fernández-Somoano, Ana Gandarillas, Pedro L. Lorenzo Ruano, Marc Marí-Dell’Olmo, Andreu Nolasco, M. Dolores Prieto-Salceda, Rebeca Ramis, Maica Rodríguez-Sanz, Pablo Sánchez-Villegas, the MEDEA3 Project Group

**Affiliations:** 1 Departament d’Estadística e Investigaciò Opertiva, Universitat de València, 46100 Burjassot, Spain; 2 CIBER de Epidemiología y Salud Pública, 28029 Madrid, Spain; corpas_fra@gva.es (F.C.-B.); zurriaga_osc@gva.es (Ó.Z.); cborrell@aspb.cat (C.B.); Lluis.Cirera@carm.es (L.C.); jdelfrao@navarra.es (J.D.O.); fernandezsana@uniovi.es (A.F.-S.); mmari@aspb.cat (M.M.-D.); rramis@isciii.es (R.R.); mrodri@aspb.cat (M.R.-S.); 3 FISABIO Foundation, 46020 Valencia, Spain; vergara_car@gva.es; 4 Dirección General de Salut Pública i Adiccions, Conselleria de Sanitat Universal y Salut Pública, 46020 Valencia, Spain; botella_pal@gva.es (P.B.-R.); perez_jorpan@gva.es (J.P.-P.); 5 Departament de Medicina Preventiva, Salut Pública, Ciències de l’Alimentación, Toxicología i Medicina Legal, Universitat de València, 46010 Valencia, Spain; 6 Dirección de Salud Pública y Adicciones, 48013 Bilbao, Spain; ealdasaro-san@euskadi.eus; 7 Agència de Salut Pública de Barcelona, 08023 Barcelona, Spain; 8 Institut d’Investigació Biomèdica (IIB Sant Pau), 08025 Barcelona, Spain; 9 Institut d’investigació sanitària de les Illes Balears, 07120 Palma de Mallorca, Spain; ecabeza@dgsanita.caib.es; 10 Department of Epidemiology, Regional Health Council-IMIB-Arrixaca, 30008 Murcia, Spain; 11 Instituto de Salud Pública y Laboral de Navarra, 31003 Pamplona, Spain; 12 IUOPA-Medicine Department, Universidad de Oviedo, 33006 Oviedo, Spain; 13 Instituto de Investigación Sanitaria del Principado de Asturias (ISPA), 33001 Oviedo, Spain; 14 Consejeria de Sanidad, 28035 Madrid, Spain; ana.gandarillas@salud.madrid.org; 15 Servicio Canario de Salud, 35018 Las Palmas de Gran Canaria, Spain; plorrua@gobiernodecanarias.org; 16 Universidad de Alicante, 03690 San Vicente del Raspeig, Spain; nolasco@ua.es; 17 Fundación Marqués de Valdecilla-Observatorio de Salud Pública de Cantabria, 39008 Santander, Spain; dprieto.ospc@fmdv.org; 18 Instituto de Salud Carlos III, 28029 Madrid, Spain; 19 Escuela Andaluza de Salud Pública, 18011 Granada, Spain; pablo.sanchez.easp@juntadeandalucia.es

**Keywords:** mortality, urban areas, geographical inequalities, multivariate disease mapping

## Abstract

The geographical distribution of mortality has frequently been studied. Nevertheless, those studies often consider isolated causes of death. In this work, we aim to study the geographical distribution of mortality in urban areas, in particular, in 26 Spanish cities. We perform an overall study of 16 causes of death, considering that their geographical patterns could be dependent and estimating the dependence between the causes of death. We study the deaths in these 26 cities during the period 1996–2015 at the census tract level. A multivariate disease mapping model is used in order to solve the potential small area estimation problems that these data could show. We find that most of the geographical patterns found show positive correlations. This suggests the existence of a transversal geographical pattern, common to most causes of deaths, which determines those patterns to a higher/lower extent depending on each disease. The causes of death that exhibit that underlying pattern in a more prominent manner are chronic obstructive pulmonary disease (COPD), lung cancer, and cirrhosis for men and cardiovascular diseases and dementias for women. Such findings are quite consistent for most of the cities in the study. The high positive correlation found between geographical patterns reflects the existence of both high and low-risk areas in urban settings, in general terms for nearly all the causes of death. Moreover, the high-risk areas found often coincide with neighborhoods known for their high deprivation. Our results suggest that dependence among causes of death is a key aspect to be taken into account when mapping mortality, at least in urban contexts.

## 1. Introduction

Geographical variability in mortality has been repeatedly studied and evidenced at very different geographical scales (nationwide, urban areas …) and levels of spatial disaggregation. Nevertheless, the spatial distribution of mortality within large cities may neither follow the same rules nor be in agreement with that same spatial distribution at other larger disaggregation levels. Just as an example, let us look at the case of Spain. Several geographical mortality studies have been undertaken in this country, some of them for the whole or part of the country at the municipal level [1,2,3,4,5] and others exploring that same variability within a number of large Spanish cities at the census tract level [6,7]. In these studies, we find causes of mortality such as Alzheimer’s disease, that display a marked geographical pattern with strong differences across the country at the municipal level for either of the two genders [5]. On the other hand, this same cause of death does not show any remarkable spatial variability when studied inside large Spanish cities at the census tract level [6,7]. In contrast, stomach cancer in men has consistently been found to be associated with deprivation at the census tract level for many large Spanish cities [8], but when this association is explored throughout the whole country, at the municipal level, it cannot be found. At the municipal level, deprivation even exhibits a protective role for stomach cancer, in contrast to what occurs in the urban context [4,5]. Taking into account the large percentage of people living in big cities presently, together with the steady increase in that percentage, there is a clear need for detailed studies of the geographical variation of mortality rates in urban areas.

Geographical mortality studies in urban areas have their own particularities. First, in this context, the use of small area estimation methods [9,10,11] is required. In contrast to country-level analyses, for example, where the units of study may be larger, detailed geographical studies in the urban context have such small (in statistical terms) units of study that it becomes necessary to use specific smoothing methods. This makes these studies technically complex from a statistical point of view, and the use of modeling tools capable of taking the maximum advantage of the data and their dependence sources is highly desirable. Second, when really small spatial units are considered in urban areas, such as census tracts, they are usually quite homogeneous in socioeconomic terms, which may help to avoid ecological fallacy. This is not necessarily the case of regional analyses covering, for example, several municipalities or counties, where the spatial units are far more heterogeneous since they are usually mixtures of people of very different social conditions and the weights of these mixtures vary for each spatial unit. Hence, regional analyses may dilute the effect of some risk factors of interest, such as deprivation or many other socioeconomic factors, making urban studies a far more suitable context for studying the effect of risk factors of this kind.

Geographical mortality studies in urban settings have often been published with different purposes. Nevertheless, many of them usually focus on some particular city and/or cause of death [12,13]. Comprehensive mortality studies covering several cities and causes of death [14,15,16] are much scarcer. These studies provide a far more general view of mortality, instead of the isolated overview of particular causes of death or cities. Moreover, more general studies covering several cities allow us to assess the extent to which the results found in one city can be replicated in relation to others. They also make it possible to discern whether these results would be something particular to a single city of study or, conversely, we would be talking instead about something general and replicable in many other cities beyond those considered.

Despite the obvious interest of the above-mentioned comprehensive urban area mortality studies for several cities, none of them, to our knowledge, have considered or explored the relationships among causes of death. Small-area models usually assume that neighboring areas are similar as a way to improve risk estimates. On the contrary, possibly due to its methodological complexity, dependence between causes of death is usually ignored, although this source of dependence could obviously improve risk estimates in a similar manner to spatial dependence. Considering that dependence could favor the spatial patterns of diseases with common risk factors, such as high tobacco or alcohol consumption, to show similar features. Interestingly, methodologies are currently being developed that allow the joint study of dozens of diseases by estimating and taking advantage of the multivariate dependence between diseases [17,18]. Considering dependence between causes of death will allow us, on the one hand, to benefit from risk estimates of that important source of dependence and, on the other hand, to estimate the multivariate dependence structure of those causes of death in urban settings.

The goal of this paper is to address the study of the geographical distribution of mortality for each sex over a large set of (26) Spanish cities and causes of death during the period 1996–2015. This will allow us to derive a comprehensive view of those causes of death and assess the replicability of our findings for the urban context in general, at least for large Spanish cities. In this study, we take census tracts as our units of analysis since their small size makes them especially well-suited for yielding insight into many risk factors, particularly social risk factors, which are generally homogeneously distributed within such tracts.

In addition, as a novelty, our final goal is to take into account, and to take advantage of, the dependence between causes of death. Estimating the dependence structure between causes of death would be an important result of our study, which would allow us to know how they relate to each other, thereby giving us valuable insight into the presence of common risk factors that they might have.

## 2. Materials and Methods

### 2.1. Design

This study was carried out within the framework of the Spanish MEDEA research project [6,19], in this case in its third edition. The aim of this project is to study the socio-economic and environmental inequalities in mortality in large Spanish cities. MEDEA is a collaborative project currently composed of 13 research groups from different regions in Spain. This study uses a cross-sectional ecological design, using data from the 1996–2015 period, and the units of analysis were the census tracts of the 26 cities included in the study. All these cities are among the 100 largest Spanish cities, according to the 2011 population census, and they include 9 out of the 10 largest cities in Spain. The population of these 26 cities comprises 25.3% of the overall Spanish population and they are located in 11 out of the 17 Spanish regions (Autonomous Communities). Therefore, they can be considered a comprehensive sample of large Spanish cities.

### 2.2. Study Population and Information Sources

The study population consisted of the people residing in the above-mentioned cities over the 20-year period of 1996–2015. Mortality data were obtained from the Spanish Statistical Institute (INE) through the regional Statistical or Health Departments or from the city mortality registry in the case of Barcelona.

The expected number of deaths in each census tract was calculated taking as a reference the mortality rates by gender, age (five-year age groups), and cause of death for each city in the study during the period 1996–2015. The numbers of inhabitants stratified by gender, age (in five-year groups), and census tract were obtained from municipal population registries. These population data, which have been publicly available since 2004, were downloaded from the INE website. The population data for the previous period, 1996–2003, were purchased from that same Institute.

### 2.3. Variables

The numbers of deaths by five-year age groups, gender, address of residence, and the underlying cause of death were extracted from mortality databases. Census tracts were generally obtained (21 out of 26 cities) through the address of the deceased, provided by the death certificate questionnaire. Those addresses were geocoded by using Cartociudad [20], a geocoding service of the Spanish National Geographical Institute, and Google Maps as an additional tool for those addresses where Cartociudad did not report a successful result. An R library, medear [21], was developed with the functions used for this task, among others, and published for public use. Using this tool enabled us to geocode 96.44% of the total number of deaths in the study. Institutional information and tools were used for the rest of the cities (5 out of 26).

Underlying causes of death were coded using the International Classification of Diseases the 9th revision (ICD-9) for deaths that occurred between 1996 and 1998, and the 10th (ICD-10) for those that occurred between 1999 and 2015. The present study analyzed mortality for 16 causes of death: Acquired Immune Deficiency Syndrome (AIDS), stomach cancer, colorectal cancer, lung cancer, breast cancer, prostate cancer, bladder cancer, hematological cancer, diabetes, dementia, ischemic heart disease (IHD), ictus, chronic obstructive pulmonary disease (COPD), cirrhosis, suicide, and traffic injuries. Some of these causes were discarded for a certain gender due to the lack of biological sense (prostate cancer in women, for example) or their low number of deaths (breast cancer in men or AIDS in women, for example). Thus, 15 causes of death were finally studied for men and 11 for women. These causes were chosen since they are either one of the leading causes of death or because of their impact in terms of years of life lost, as is the case of AIDS, suicides, and traffic injuries. A table with the ICD codes and number of deaths for each cause of death is supplied as supplementary material to the paper. These causes of death accounted for 47.6% of all deaths among men and 34.9% of those among women in Spain for the year 2011. All variables and analyses in this study were considered separately for each of the sexes.

### 2.4. Data Analysis

Smoothed Standardized Mortality Ratios (SMRs) were used as summaries of the risk of mortality. These SMRs were derived as the relationship between observed and expected deaths for each census tract and cause of death according to the following model. A Bayesian multivariate spatial model was used for each city to overcome the small area estimation problem underlying our dataset. Specifically, for a given city of study, if O_ij_ and E_ij_ denote, respectively, the observed and (age-standardized) expected deaths in census tract i and cause of death j, then O_ij_ is assumed to follow a Poisson distribution of mean θ_ij_⋅E_ij_. The SMRs followed would simply be equal to SMR_ij_ = 100⋅θ_ij_. The matrix θ = (θ_ij_) is modeled as θ = φ⋅M, where the columns of φ follow independent spatial Leroux’s distributions [22] and the cells of M follow independent normal distributions. In this way, φ induces spatial dependence in the estimates of the SMRs and M induces multivariate dependence between diseases. Specifically, our modeling proposal induces the following variance-covariance matrix between diseases Σ = M’⋅M [17], which is also estimated within the model and allows taking advantage of dependence between diseases if this really existed. However, if one disease was independent of the rest, this could be also reproduced by this model, making the off-diagonal elements of the corresponding row and column of Σ equal to 0. Spatial and multivariate dependence is expected to improve the risk estimates and to overcome the small area estimation problems associated with such disaggregated data.

This modeling proposal belongs to what are known as M-models, which have already been used for the joint study of 21 causes of death [17]. Nevertheless, during the set-up of this project, we improved the original M-model proposals by allowing them to explicitly consider different spatial variances for each cause of death [23] and by estimating a suitable spatial weights matrix for our dataset [24]. The final statistical modeling proposal in our study includes these two improvements.

The inference was performed using Markov chain Monte Carlo methods (MCMC) by means of WinBUGS, version 1.4.3 [25]. R 3.1 [26] and, in particular, the pbugs library [27], which allowed us to speed up the MCMC computation by parallelizing its different chains, were used for data management and posterior analysis of the WinBUGS results. Model convergence was assessed using the R-hat Brooks–Gelman–Rubin statistic [28] and effective sample size of the chains [29]. Criteria for convergence were: R-hat less than 1.1 and effective sample size greater than 100 for all the identifiable parameters retrieved from the model. In order to enhance the reproducibility of our study, an Rmarkdown file with the code used for the statistical analysis of the paper, and the data required to run it, are supplied also as Supplementary Material.

Regarding the particular statistical analysis of this paper, in addition to the above-mentioned statistical analysis used to derive the SMRs for the whole MEDEA project, we also used two-way ANOVA to assess the effect of the city under study and the cause of death on the spatial variability found for each combination of these two factors. We also used a slightly tuned version of the corrplot function of the R library of the same name [30], and Pearson’s correlation coefficients, in order to generate the correlation plots shown in the Results section. Choropleth maps were drawn in order to show the geographical distribution of risks for some causes of death and cities.

Finally, we developed and published a web app http://www.uv.es/medea/medeapp.html (accessed on 24 May 2021) that allows the geographic information generated within the MEDEA project to be explored in detail. This tool allows navigation and exploration of the SMR maps for each city and cause of death in the study. Furthermore, the app contains a number of additional tools, such as maps of the significance of the risk excesses, that allow interested users to take advantage of the information generated within the project. This web app was developed using the Shiny R package [31].

## 3. Results

Table 1 and Table 2 show, for each cause of death, the standard deviation of the (log-)SMRs throughout the census tracts of the corresponding city. Additionally, these tables include a final row and column, respectively, with the average standard deviation for each city (for the collection of causes of death considered) and the average standard deviation for each cause of death (for the collection of cities in the study). These tables quantify for men and women, respectively, the spatial variability of each cause of death and city, that is, the strength of the geographical inequalities that we are seeing. In the case of men, the largest differences in geographical mortality are observed for AIDS, in particular in Cordoba, Sevilla, and Granada, all three cities located in southern Spain. In contrast, prostate cancer is the cause of death showing the most homogeneous spatial distributions, in particular over Alicante, Oviedo, and Valencia. Similarly, for women, diabetes displayed the most variable spatial patterns, in particular in San Sebastián, Palma de Mallorca, and Murcia, while hematological cancer, in particular in Santander and Gijón, showed the most evenly distributed risk patterns.

For Table 1 and Table 2 we assessed the effect of the city and cause of death on the spatial variability (standard deviations) of the corresponding combinations of factors. Thus, for men, we observed that both the cause of death (*p*-value < 2 × 10^−16^) and the city (*p*-value = 1.9 × 10^−15^) have a significant effect on the strength of the spatial risk patterns found. For women, we also found that both factors had a significant effect (*p*-value < 2 × 10^−16^ for the cause of death and 3.0 × 10^−12^ for the city). This result points towards the existence of important risk factors that make some cities report more pronounced mortality inequalities than others although, interestingly, differences among causes of death seem to be even stronger among cities.

The causes of death in Table 1 and Table 2 are ordered by their average variability for the set of cities considered. Thus, for men, the causes of death showing the largest spatial variabilities are AIDS, cirrhosis, and COPD, in that order, whereas those with the smallest variabilities are prostate, hematological, and colorectal cancer. For women, diabetes, dementia, and IHD display the largest variability whereas hematological, colorectal, and breast cancers have the smallest variabilities. For those causes of death studied for both genders, the variabilities found for men and women yielded a correlation of 0.38, indicating that those causes of death that have a marked spatial pattern for one of the genders also tend to show a variable spatial pattern for the other gender.

**Figure 1:**
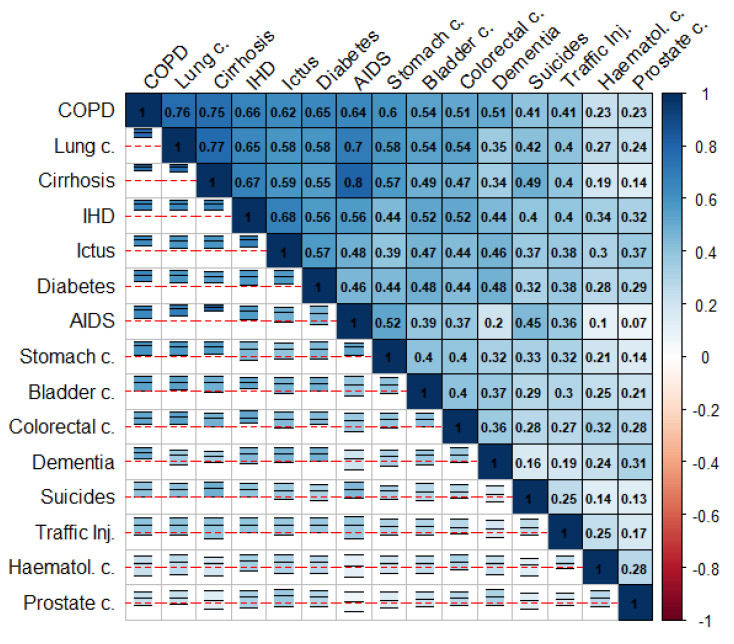
Mean (log)-SMRs correlations between causes of death (upper triangle) and 95% interval for the observed correlation across cities (lower triangle) in men.

**Figure 2:**
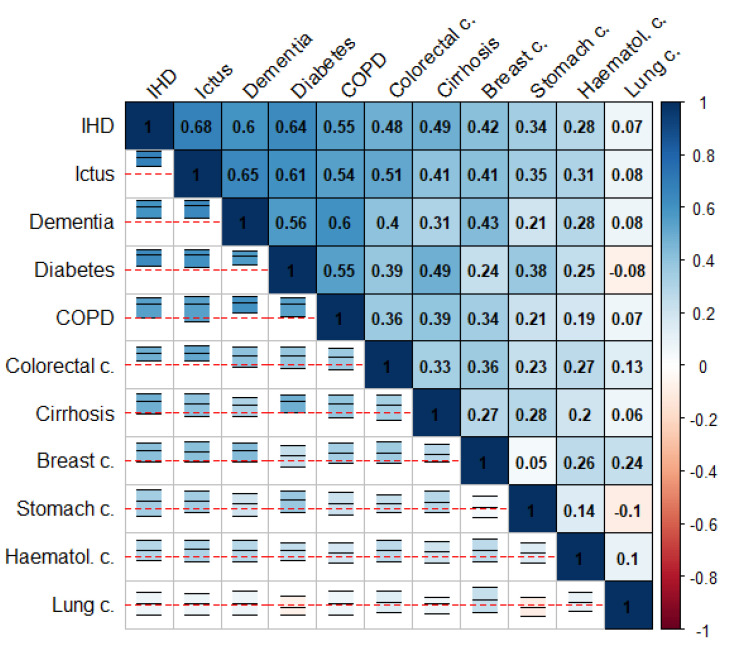
Mean (log-)SMRs correlations between causes of death (upper triangle) and 95% interval for the observed correlation across cities (lower triangle) in women.

Figure 1 and Figure 2 summarize the correlation structure between causes of death for the collection of cities in the study as a whole. This figure displays (upper triangle), for each pair of causes of death, the mean correlation for the (log-)SMRs for the cities in the study. Similarly, specific correlation matrices for each of the cities in the study can be found on the “Spatial analysis > Additional information” tab of the web app of the project. Note that the causes of death in these figures were ordered according to their mean correlations with the rest of the causes of death. The lower triangle in these figures shows intervals delimiting the 2.5 and 97.5 percentiles for the corresponding correlations for all the cities in the study. The red horizontal lines correspond to 0, so these intervals allow assessment of the variability among cities for these correlations. We can, therefore, see how COPD and lung cancer in men or IHD and ictus in women exhibit positive correlations consistently for at least 95% of the cities in the study. This is something quite general for the causes of death on the top-left side of Figure 1 and Figure 2. In contrast, for prostate cancer and AIDS in men, we find a similar number of cities offering either positive or negative correlations. This is the general case of the causes of death on the bottom-right side of Figure 1 and Figure 2.

These figures show that the pairwise correlations for most of the spatial patterns found are positive. This means that, in general, we find sets of census tracts where most of the causes of death exhibit risk excesses whereas, on the contrary, there are other regions where most of the causes of death have low risks compared to the rest of the city. That is, overall, in mortality terms, we find protective and high-risk regions and these regions usually coincide for many causes of death. Interestingly, the causes of death on the upper-left side of Figure 1 and Figure 2 coincide in general with those of higher spatial variability in Table 1 and Table 2. In particular, for men, COPD, lung cancer, and cirrhosis (top-left causes in Figure 1) are, respectively the third, fourth, and second causes of death according to their spatial variability, while prostate cancer, hematological cancer, and traffic injuries (bottom-right causes in Figure 1) are, respectively the 15th, 14th, and 11th (out of 15) causes of death according to this criterion. For women, we find something similar since IHD, ictus and dementia are the third, fifth, and second causes according to their spatial variability while lung, hematological and stomach cancers are the 8th, 11th and 7th (out of 11) causes of death according to this criterion.

Figure 1 and Figure 2 also show some interesting differences between men and women. Thus, the mean correlation between any pair of diseases is higher in men than in women, 0.41 vs. 0.32 (t-test *p*-value for mean differences: 0.007). This suggests a greater agreement for the spatial mortality patterns in men than in women. Interestingly, correlations between spatial patterns for women are even negative in some cases, as for lung cancer and diabetes or lung and stomach cancer. This does not happen in men, who always show positive correlations between spatial patterns.

**Figure 3:**
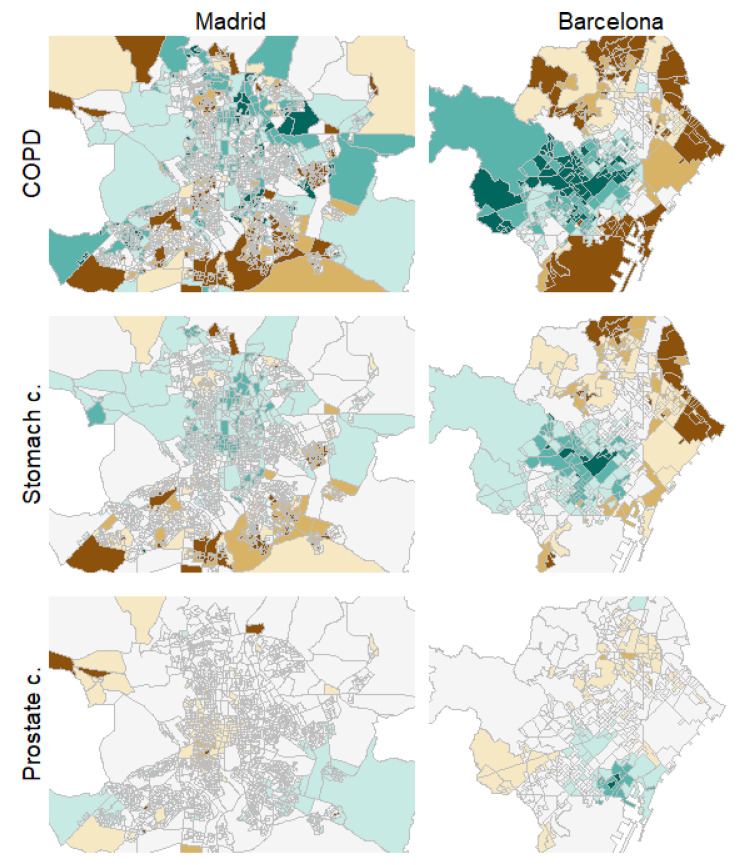
Choropleth maps for COPD in men, stomach and prostate cancer mortality risk patterns in men for the cities of Madrid (**left**) and Barcelona (**right**).

Figure 3 shows, as an example, the estimated SMRs for COPD, stomach, and prostate cancer mortality in men for the cities of Madrid and Barcelona, the two largest cities included in the study. These three causes of death are meant to represent causes with high, middle, and low spatial correlations, respectively, with the rest of the causes. Brown colors denote high-risk areas in these maps and green colors protective areas, while darker colors mean more extreme SMRs. Fixed risk levels, with cuts equal to 0.7, 0.8, 0.9, 1.1, 1.2, and 1.3, were used for all the maps. A progressive reduction in spatial variability can be observed for these three diseases and for the two cities. Additionally, also for both cities, we find how the spatial patterns for COPD and stomach cancer are quite similar although, obviously, each of them may exhibit specific particularities.

Similar results to those shown in Figure 3 (for example, similar spatial patterns for the causes of death placed on the upper-left side of Figure 1 and Figure 2) may be found for the rest of the cities in the study. These spatial patterns can be found and explored in full detail under the “Spatial analysis” tab of the project website. Additionally, maps with the probability of risk excess for the census tracts of these cities and for the different causes of death, among other results, may also be found and explored in full detail on that website.

## 4. Discussion

Although mortality studies of several causes of death had previously been performed in urban settings, in this paper, we pay particular attention to how these causes of death relate to each other. To our knowledge, this approach has not yet been taken in the small area analysis literature. As a result, in addition to the improved smoothed SMR estimates that we have generated, we were also able to derive an important tool in the form of the correlation matrix between diseases that sheds light on how the geographical distributions of these causes of death are related. Additionally, our approach allowed us to characterize and display the performance of regions with a particularly high risk for a given cause of death. Specifically, we addressed whether a region that exhibits a high risk for one cause of death also has a high risk for the rest or, in contrast, competitive risks emerge, thereby decreasing the risk for the other causes. We found that, in general, when a region displays a high risk for some of the causes of death, we also find risk excesses for the rest of the diseases in that same region. We also found that this will be more likely when the causes of death that we are talking about exhibit a prominent spatial variability. We, therefore, showed that when the spatial variability is lower, the corresponding cause of death usually displays a more erratic spatial pattern that does not resemble so much the pattern of the other causes of death.

In addition, many of the high-risk areas shown for COPD and stomach cancer in Figure 3, which are representative of the high-risk areas for most of the causes of death studied here, belong to neighborhoods known for their high levels of deprivation, such as the north (Tetuán), east (San Blas) and south-east (Puente de Vallecas) high-risk clusters in Madrid or the north-east (Ciutat Meridiana-Torre Baró) and east (El Besòs i el Maresme) high risk clusters in Barcelona. This issue can be confirmed by the “Socioeconomic analysis > Deprivation index” tab of the website of the project, where a deprivation index at the census tract level can be found for all the cities included in the study. Thus, according to these results, a strong association between deprivation and mortality risk excesses seems to arise when spatial variability is more relevant. This same association can also be noticed for the rest of the cities on the project website.

As a consequence of Figure 1 and Figure 2, in general in urban settings, we find regions with either good or bad performance in terms of mortality. If one census tract displays high/low risks for a cause of death, it also tends to show similar performance for the rest of the diseases. It, therefore, seems as if most causes of mortality had one or several spatially correlated determinant(s) that affect mortality in general, although with varying strengths depending on the different causes of death. Evidently, additional risk factors could be determining the particular spatial pattern of mortality for some causes of death, but they will only be modifying the main spatial pattern that we are finding to be common to most of the causes of death in this paper. Additionally, although this should be confirmed by a specific ecological regression study, high-risk mortality regions seem to coincide with highly deprived regions. This would suggest that deprivation could be the main determinant of mortality, in general terms, although this factor could have different strengths for each cause of death. There are abundantly documented associations between neighborhood socioeconomic characteristics and risk for specific causes of death [32,33]. In this study, as shown, when the spatial patterns found do not correlate with deprivation, they exhibit milder spatial variability.

Other studies [34] have shown that the impact of deprivation on mortality in small areas is sustained over time, moreover, a significant statistical association is found between all the causes of death in that study and deprivation. In the deprived areas, the impact on mortality is sometimes attributed to their physical and social environments [35], but neighborhood disadvantage and economic stress also may impact individual mortality [36] independently from individual socioeconomic characteristics. Other common factors, such as tobacco, alcohol consumption, whose spatial distribution could be similar to that of deprivation, could be also determining the spatial patterns emerging from this study, or even the evidenced association between spatial patterns. Nevertheless, other not-so-evident risk factors, such as unhealthy food environments [37], heatwave vulnerability [38,39], traffic noise [40,41], or environmental pollution [42], often correlated with deprivation, could be increasing mortality risks in deprived areas for some causes of death, making deprivation a multifaceted construct that merges many separate risk factors in addition to socioeconomic features.

Our study also shows important differences between sexes in the spatial distribution of mortality. Thus, we show how mortality in men shows a more clustered geographical pattern than in women, with a higher geographical agreement for all causes of death. Interestingly, the web app of the project shows all the spatial patterns estimated in this project and it can be seen that mortality in men cluster, in general, around the highly deprived areas to a larger extent than women. This suggests that deprivation could be playing a different role for both sexes, men being more sensible to deprivation in terms of mortality than women [43,44].

One of the main strengths of this study is its dimensions. The region of study comprises a population of 11.7 million people in the mid-year of study (2005). In addition, the period of study covers 20 years and 16 causes of death, all of which are interrelated. These features should make the results of the study quite solid and powerful, in statistical terms. Moreover, a total of 26 large cities were considered in the study. This makes it possible to formulate our results in general terms, and to assess their validity for additional new cities, instead of them being particular findings regarding certain mortality patterns for some specific city.

Regarding the limitations of the study, we find that its ecological nature could be seen as a drawback. Nevertheless, in our opinion, the small size of census tracts is another of the strengths of the study, as it makes them quite homogeneous in socioeconomic terms, thereby limiting the potential problems of the ecological character of the study. Additionally, the goal of this study, namely, exploring how mortality risks are related for different causes, can only be addressed under an ecological perspective since, individually, people show just one of the causes of the study, which prevents us from achieving this goal from an individual approach.

Another drawback of this work is the (lack of) the assessment of the impact that some confounding factors, or determinants, could be having on the geographical distributions of mortality emerging from this study. Regretfully, the lack of information on risk determinants such as tobacco or alcohol consumption, at the census tract level, has prevented us from exploring their effects. A careful assessment of the effect of these factors, on both the geographical patterns of mortality and their correlations, in an urban context, is an interesting line of research to be explored in future studies.

## 5. Conclusions

This study shows that, in urban settings, when spatial variability in mortality is high for one cause, that spatial pattern is often similar to that of most of the rest of the mortality causes. This suggests the convenience of multivariate geographical mortality analyses of several causes of death, at least in urban studies, over isolate studies of just one disease. The latest of these options could be missing the confounding effect of the overall spatial mortality pattern, which is common to most of the causes of death, making us interpret this pattern as a particular feature of the disease of study instead of a more general setting. This could possibly lead us to misleading conclusions. In addition, the high dependence between the diseases evidenced suggests that actions reverting mortality inequalities should be taken following an overall approach, which takes all mortality causes into account instead of isolated actions for some particular cause of death. This new overall view of geographical mortality studies is possibly the main conclusion of this work.

## Figures and Tables

**Table 1:** Variability (standard deviation) of the (log-)SMRs for each cause of death and city in men.

	AIDS	Cirrhosis	COPD	Lung c.	Diabetes	IHD	Dementia	Ictus	Stomach c.	Suicides	Traffic Inj.	Bladder c.	Colorectal c.	Haematol. c.	Prostate c.	Mean
Alicante	0.52	0.29	0.25	0.14	0.15	0.12	0.10	0.12	0.13	0.26	0.10	0.15	0.02	0.06	0.03	0.16
Almería	0.69	0.40	0.22	0.21	0.35	0.18	0.10	0.13	0.19	0.14	0.17	0.15	0.16	0.11	0.05	0.22
Avilés	0.20	0.30	0.27	0.14	0.17	0.10	0.21	0.15	0.11	0.14	0.07	0.20	0.06	0.15	0.22	0.16
Barcelona	0.48	0.25	0.30	0.14	0.17	0.08	0.21	0.10	0.19	0.13	0.10	0.11	0.11	0.07	0.10	0.17
Bilbao	0.65	0.26	0.31	0.18	0.17	0.12	0.15	0.11	0.17	0.18	0.04	0.08	0.10	0.08	0.09	0.18
Cádiz	0.52	0.25	0.22	0.19	0.10	0.17	0.08	0.13	0.07	0.04	0.20	0.04	0.09	0.17	0.09	0.16
Cartagena	0.46	0.29	0.25	0.20	0.13	0.18	0.17	0.20	0.12	0.11	0.09	0.19	0.14	0.14	0.11	0.19
Castellón	0.32	0.18	0.14	0.09	0.05	0.08	0.04	0.08	0.12	0.12	0.09	0.07	0.05	0.08	0.05	0.10
Córdoba	0.84	0.31	0.25	0.18	0.21	0.18	0.16	0.17	0.13	0.11	0.09	0.18	0.09	0.11	0.16	0.21
Gijón	0.19	0.19	0.19	0.13	0.08	0.11	0.08	0.08	0.10	0.06	0.07	0.07	0.08	0.06	0.05	0.10
Granada	0.70	0.35	0.34	0.19	0.18	0.24	0.13	0.18	0.18	0.21	0.21	0.22	0.21	0.07	0.14	0.24
Huelva	0.68	0.32	0.39	0.25	0.23	0.18	0.27	0.25	0.13	0.11	0.15	0.18	0.19	0.15	0.17	0.24
Jaén	0.34	0.26	0.21	0.12	0.06	0.15	0.06	0.19	0.13	0.20	0.07	0.08	0.07	0.12	0.09	0.14
Madrid	0.61	0.31	0.23	0.16	0.15	0.12	0.16	0.13	0.15	0.12	0.10	0.09	0.08	0.05	0.09	0.17
Málaga	0.57	0.23	0.25	0.17	0.21	0.16	0.15	0.12	0.09	0.09	0.16	0.11	0.07	0.04	0.09	0.17
Murcia	0.37	0.29	0.25	0.18	0.12	0.10	0.16	0.15	0.12	0.08	0.15	0.06	0.05	0.05	0.12	0.15
Oviedo	0.35	0.20	0.20	0.17	0.13	0.11	0.09	0.15	0.17	0.11	0.05	0.17	0.11	0.07	0.03	0.14
Palma de Mallorca	0.35	0.17	0.29	0.13	0.19	0.15	0.25	0.12	0.15	0.17	0.09	0.10	0.05	0.06	0.08	0.16
Palmas Gran Canaria	0.40	0.34	0.32	0.21	0.20	0.18	0.06	0.18	0.24	0.18	0.24	0.14	0.07	0.09	0.07	0.20
Pamplona	0.37	0.16	0.22	0.14	0.15	0.18	0.23	0.11	0.08	0.04	0.07	0.12	0.06	0.14	0.06	0.14
San Sebastián	0.35	0.25	0.21	0.14	0.15	0.12	0.18	0.11	0.09	0.05	0.07	0.05	0.07	0.10	0.08	0.13
Santa Cruz Tenerife	0.50	0.24	0.34	0.21	0.21	0.15	0.12	0.08	0.14	0.08	0.19	0.09	0.09	0.08	0.18	0.18
Santander	0.17	0.17	0.27	0.12	0.12	0.12	0.15	0.15	0.09	0.10	0.07	0.07	0.12	0.07	0.04	0.12
Sevilla	0.74	0.39	0.32	0.21	0.16	0.15	0.11	0.17	0.15	0.22	0.20	0.15	0.10	0.06	0.08	0.21
Valencia	0.55	0.22	0.19	0.12	0.11	0.11	0.09	0.10	0.10	0.04	0.12	0.10	0.04	0.07	0.03	0.13
Vitoria	0.56	0.37	0.25	0.14	0.08	0.19	0.15	0.15	0.05	0.20	0.12	0.10	0.11	0.13	0.06	0.18
Mean	0.48	0.27	0.26	0.16	0.16	0.14	0.14	0.14	0.13	0.13	0.12	0.12	0.09	0.09	0.09	

**Table 2:** Variability (standard deviation) of the (log-)SMRs for each cause of death and city in women.

	Diabetes	Dementia	IHD	COPD	Ictus	Cirrhosis	Stomach c.	Lung c.	Breast c.	Colorectal c.	Haematol. c.	Mean
Alicante	0.16	0.21	0.13	0.13	0.10	0.19	0.04	0.06	0.07	0.04	0.05	0.11
Almería	0.19	0.20	0.27	0.08	0.18	0.05	0.08	0.11	0.10	0.14	0.10	0.14
Avilés	0.05	0.24	0.10	0.11	0.22	0.07	0.02	0.05	0.15	0.05	0.06	0.10
Barcelona	0.26	0.24	0.19	0.27	0.15	0.21	0.13	0.20	0.14	0.10	0.10	0.18
Bilbao	0.19	0.23	0.10	0.18	0.08	0.21	0.22	0.16	0.04	0.06	0.03	0.14
Cádiz	0.15	0.10	0.19	0.07	0.11	0.16	0.13	0.08	0.08	0.07	0.14	0.12
Cartagena	0.19	0.18	0.20	0.24	0.23	0.20	0.07	0.05	0.18	0.17	0.11	0.16
Castellón	0.07	0.15	0.10	0.06	0.09	0.05	0.04	0.10	0.02	0.07	0.04	0.07
Córdoba	0.17	0.20	0.21	0.18	0.17	0.12	0.20	0.09	0.10	0.09	0.12	0.15
Gijón	0.17	0.13	0.15	0.13	0.12	0.04	0.11	0.05	0.09	0.06	0.02	0.10
Granada	0.29	0.14	0.20	0.16	0.21	0.26	0.20	0.07	0.09	0.11	0.09	0.17
Huelva	0.26	0.27	0.20	0.18	0.20	0.14	0.07	0.12	0.10	0.12	0.08	0.16
Jaén	0.27	0.17	0.16	0.25	0.12	0.22	0.27	0.07	0.04	0.13	0.09	0.16
Madrid	0.21	0.24	0.15	0.22	0.13	0.12	0.11	0.20	0.08	0.08	0.05	0.14
Málaga	0.22	0.25	0.14	0.11	0.15	0.16	0.11	0.06	0.07	0.09	0.04	0.13
Murcia	0.33	0.24	0.17	0.17	0.19	0.14	0.12	0.11	0.09	0.10	0.11	0.16
Oviedo	0.24	0.10	0.11	0.11	0.09	0.09	0.16	0.06	0.04	0.04	0.04	0.10
Palma de Mallorca	0.34	0.40	0.25	0.23	0.21	0.10	0.15	0.13	0.17	0.12	0.08	0.20
Palmas Gran Canaria	0.31	0.10	0.19	0.22	0.18	0.14	0.18	0.07	0.08	0.09	0.14	0.16
Pamplona	0.21	0.25	0.19	0.21	0.16	0.08	0.05	0.05	0.06	0.12	0.05	0.13
San Sebastián	0.34	0.31	0.17	0.24	0.17	0.19	0.18	0.07	0.07	0.06	0.08	0.17
Santa Cruz Tenerife	0.20	0.08	0.13	0.09	0.09	0.17	0.02	0.10	0.09	0.05	0.05	0.10
Santander	0.19	0.22	0.17	0.17	0.19	0.09	0.05	0.05	0.10	0.08	0.02	0.12
Sevilla	0.27	0.20	0.22	0.20	0.22	0.31	0.17	0.12	0.12	0.10	0.08	0.18
Valencia	0.17	0.12	0.09	0.13	0.10	0.14	0.06	0.10	0.06	0.08	0.07	0.10
Vitoria	0.14	0.23	0.22	0.21	0.10	0.06	0.05	0.07	0.09	0.08	0.08	0.12
Mean	0.21	0.20	0.17	0.17	0.15	0.14	0.11	0.09	0.09	0.09	0.07	

## Data Availability

The smoothed Standardized Mortality Ratios used in this paper, and all the data required to reproduce its analyses, are provided as supplementary material. Raw data used for deriving those estimates are not provided for privacy issues, given the small size of the units of study.

## Supplementary Materials

The following are available online at https://www.mdpi.com/article/10.3390/ijerph18115664/s1. Table S1: Causes of death considered for the MEDEA3 project. Supplementary file 1: Rmarkdown document with the code used for statistical analysis. Supplementary file 2: Data required to run Supplementary file 1.
