# Supplementary material for: Geographical Variability in Mortality in Urban Areas: A Joint Analysis of 16 Causes of Death
Source: Int J Environ Res Public Health. 2021 May 25;18(11):5664. doi: 10.3390/ijerph18115664 (PMC8197960; doi:10.3390/ijerph18115664)
Supplement: Supplementary file 1 [file ijerph-18-05664-s001.zip › Supplementary file 1.pdf]

Supplementary material to the paper 'Geographical variability en mortality in large Spanish cities: a multivariate study of 16 causes of death'

R Code used for the statistical analysis of the paper

1. Setup

```
knitr::opts_chunk$set(echo = TRUE, message = FALSE, warning=FALSE)

library(flextable)
library(officer)
library(dplyr)

##
## Attaching package: 'dplyr'

## The following objects are masked from 'package:stats':
##
##     filter, lag

## The following objects are masked from 'package:base':
##
##     intersect, setdiff, setequal, union

library(corrplot)

## corrplot 0.84 loaded

library(sf)

## Linking to GEOS 3.8.0, GDAL 3.0.4, PROJ 6.3.1

library(RColorBrewer)

# This is a slightly tunned version of the corrplot::crorrplot function
source("corrplot_plotCI.R")

# Cities information
medea3_cities <- readRDS("data/ciudades_medea3.rds")
# Cities abbreviations
medea3_cities$nombre_municipio[c(1, 2, 7, 10, 15, 16, 17, 21, 22)] <-
c("Vitoria", "Alicante", "Castellón", "San Sebastián", "Murcia", "Cartagena",
"Pamplona", "Palmas Gran Canaria", "Santa Cruz Tenerife")

# Smoothed SMRs for each cause of death and city
res_finales_sp <- readRDS("data/res_finales_sp.rds")
```

```r
# Correlation matrices derived from the multivariate spatial model
matcor_sp <- readRDS("data/matcor_sp.rds")

# Cartography
carto_medea3_sf <- readRDS("data/carto_medea3_sf.rds")

# Causes of Death
Causes <- list()
Causes$Men <- c("AIDS", "Stomach c.", "Colorectal c.", "Lung c.", "Prostate
c.", "Bladder c.", "Haematological c.", "Diabetes", "Dementia", "IHD",
"Ictus", "COPD", "Cirrhosis", "Suicides", "Traffic Inj.")
Causes$Women <- c("Stomach c.", "Colorectal c.", "Lung c.", "Breast c.",
"Haematological c.", "Diabetes", "Dementia", "IHD", "Ictus", "COPD",
"Cirrhosis")
```

2. Standard deviations analysis

```r
# log(SMRs) standard deviation for each cause of death and city
desv_log_SMRs <- list()

for(sex in 1:2){
  desv_log_SMRs[[sex]] <- matrix(NA, nrow = nrow(medea3_cities), ncol =
dim(res_finales_sp[[sex]])[2])
  rownames(desv_log_SMRs[[sex]]) <- medea3_cities$nombre_municipio
  colnames(desv_log_SMRs[[sex]]) <- Causes[[sex]]
  for(city in 1:nrow(medea3_cities)){
    codigo <- strsplit(medea3_cities$codigo[city], "-")[[1]]
    desv_log_SMRs[[sex]][city,] <-
apply(res_finales_sp[[sex]][which(substr(rownames(res_finales_sp[[sex]][,,"RM
Es"]), 1, 5)%in%codigo),,"RMEs"], 2, function(x) sd(log(x)))
  }

  # Cities are ordered alphabetically and cause od according to their
variability
  desv_log_SMRs[[sex]] <-
desv_log_SMRs[[sex]][order(rownames(desv_log_SMRs[[sex]])),]
  desv_log_SMRs[[sex]] <-
desv_log_SMRs[[sex]][,order(apply(desv_log_SMRs[[sex]], 2, mean), decreasing
= TRUE)]
}

names(desv_log_SMRs) <- c("Men", "Women")
```

```r
# Men
mean_disease <- apply(desv_log_SMRs$Men, 2, mean)
mean_city <- apply(desv_log_SMRs$Men, 1, mean)

Tabla <- as.data.frame(rbind(cbind(desv_log_SMRs$Men, Mean = mean_city), Mean = c(mean_disease, NA)))
Tabla <- cbind(c(rownames(desv_log_SMRs$Men), "Mean"), Tabla)
colnames(Tabla)[1] <- " "

h_header <- max(dim_pretty(flextable(Tabla), part = "header")$widths)
Tabla <- flextable(Tabla) %>%
  colformat_num(digits = 2) %>%
    vline(j = c('Prostate c.'), border = fp_border(), part = "all") %>%
      hline(i = 26, border = fp_border()) %>%
          rotate(j = 1:dim(Tabla)[2], rotation = "btlr", part = "header") %>%
            height(i = 1, h_header-0.4, part = "header") %>%
              hrule(i = 1, rule = "exact", part = "header") %>%
                width(j = 1: ncol(Tabla), width = c(1.4, rep(0.4, ncol(Tabla)-1))) %>%
                  fontsize(size = 8.5, part = "all") %>%
                    set_caption(caption = "Variability (standard deviation) of the log(SMRs) for each
disease in men and city.", autonum = run_autonum(pre_label = "Table ", post_label = ". "))


Tabla
```

Table 1. Variability (standard deviation) of the log(SMRs) for each disease in men and city.

	AIDS	Cirrhosis	COPD	Lung c.	Diabetes	IHD	Dementia	Ictus	Stomach c.	Suicides	Traffic Inj.	Bladder c.	Colorectal c.	Haematological c.	Prostate c.	Mean
Alicante	0.52	0.29	0.25	0.14	0.15	0.12	0.10	0.12	0.13	0.26	0.10	0.15	0.02	0.06	0.03	0.16
Almería	0.69	0.40	0.22	0.21	0.35	0.18	0.10	0.13	0.19	0.14	0.17	0.15	0.16	0.11	0.05	0.22
Avilés	0.20	0.30	0.27	0.14	0.17	0.10	0.21	0.15	0.11	0.14	0.07	0.20	0.06	0.15	0.22	0.16
Barcelona	0.48	0.25	0.30	0.14	0.17	0.08	0.21	0.10	0.19	0.13	0.10	0.11	0.11	0.07	0.10	0.17

	AIDS	Cirrhosis	COPD	Lung c.	Diabetes	IHD	Dementia	Ictus	Stomach c.	Suicides	Traffic Inj.	Bladder c.	Colorectal c.	Haematological c.	Prostate c.	Mean
Bilbao	0.65	0.26	0.31	0.18	0.17	0.12	0.15	0.11	0.17	0.18	0.04	0.08	0.10	0.08	0.09	0.18
Cádiz	0.52	0.25	0.22	0.19	0.10	0.17	0.08	0.13	0.07	0.04	0.20	0.04	0.09	0.17	0.09	0.16
Cartagena	0.46	0.29	0.25	0.20	0.13	0.18	0.17	0.20	0.12	0.11	0.09	0.19	0.14	0.14	0.11	0.19
Castellón	0.32	0.18	0.14	0.09	0.05	0.08	0.04	0.08	0.12	0.12	0.09	0.07	0.05	0.08	0.05	0.10
Córdoba	0.84	0.31	0.25	0.18	0.21	0.18	0.16	0.17	0.13	0.11	0.09	0.18	0.09	0.11	0.16	0.21
Gijón	0.19	0.19	0.19	0.13	0.08	0.11	0.08	0.08	0.10	0.06	0.07	0.07	0.08	0.06	0.05	0.10
Granada	0.70	0.35	0.34	0.19	0.18	0.24	0.13	0.18	0.18	0.21	0.21	0.22	0.21	0.07	0.14	0.24
Huelva	0.68	0.32	0.39	0.25	0.23	0.18	0.27	0.25	0.13	0.11	0.15	0.18	0.19	0.15	0.17	0.24
Jaén	0.34	0.26	0.21	0.12	0.06	0.15	0.06	0.19	0.13	0.20	0.07	0.08	0.07	0.12	0.09	0.14
Madrid	0.61	0.31	0.23	0.16	0.15	0.12	0.16	0.13	0.15	0.12	0.10	0.09	0.08	0.05	0.09	0.17
Málaga	0.57	0.23	0.25	0.17	0.21	0.16	0.15	0.12	0.09	0.09	0.16	0.11	0.07	0.04	0.09	0.17
Murcia	0.37	0.29	0.25	0.18	0.12	0.10	0.16	0.15	0.12	0.08	0.15	0.06	0.05	0.05	0.12	0.15
Oviedo	0.35	0.20	0.20	0.17	0.13	0.11	0.09	0.15	0.17	0.11	0.05	0.17	0.11	0.07	0.03	0.14
Palma de Mallorca	0.35	0.17	0.29	0.13	0.19	0.15	0.25	0.12	0.15	0.17	0.09	0.10	0.05	0.06	0.08	0.16
Palmas Gran Canaria	0.40	0.34	0.32	0.21	0.20	0.18	0.06	0.18	0.24	0.18	0.24	0.14	0.07	0.09	0.07	0.20
Pamplona	0.37	0.16	0.22	0.14	0.15	0.18	0.23	0.11	0.08	0.04	0.07	0.12	0.06	0.14	0.06	0.14
San Sebastián	0.35	0.25	0.21	0.14	0.15	0.12	0.18	0.11	0.09	0.05	0.07	0.05	0.07	0.10	0.08	0.13
Santa Cruz Tenerife	0.50	0.24	0.34	0.21	0.21	0.15	0.12	0.08	0.14	0.08	0.19	0.09	0.09	0.08	0.18	0.18
Santander	0.17	0.17	0.27	0.12	0.12	0.12	0.15	0.15	0.09	0.10	0.07	0.07	0.12	0.07	0.04	0.12
Sevilla	0.74	0.39	0.32	0.21	0.16	0.15	0.11	0.17	0.15	0.22	0.20	0.15	0.10	0.06	0.08	0.21
Valencia	0.55	0.22	0.19	0.12	0.11	0.11	0.09	0.10	0.10	0.04	0.12	0.10	0.04	0.07	0.03	0.13
Vitoria	0.56	0.37	0.25	0.14	0.08	0.19	0.15	0.15	0.05	0.20	0.12	0.10	0.11	0.13	0.06	0.18
Mean	0.48	0.27	0.26	0.16	0.16	0.14	0.14	0.14	0.13	0.13	0.12	0.12	0.09	0.09	0.09	

```r
# log(SMRs) standard deviations comparison as a function of cause of death
and city

# Men
sex <- 1
City <- rep(medea3_cities$codigo, dim(desv_log_SMRs[[sex]])[2])
Disease <- rep(colnames(desv_log_SMRs[[sex]]), each =
dim(desv_log_SMRs[[sex]])[1])
Variability <- c(desv_log_SMRs[[sex]])
data_fit <- data.frame(City, Disease, Variability)
fit_anova <- aov(Variability ~ Disease + City, data = data_fit)
print(summary(fit_anova))

# Women
mean_disease <- apply(desv_log_SMRs$Women, 2, mean)
mean_city <- apply(desv_log_SMRs$Women, 1, mean)

Tabla <- as.data.frame(rbind(cbind(desv_log_SMRs$Women, Mean = mean_city),
Mean = c(mean_disease, NA)))
Tabla <- cbind(c(rownames(desv_log_SMRs$Women), "Mean"), Tabla)
colnames(Tabla)[1] <- " "

h_header <- max(dim_pretty(flextable(Tabla), part = "header")$widths)
Tabla <- flextable(Tabla) %>%
  colformat_num(digits = 2) %>%
    vline(j = c('Haematological c.'), border = fp_border(), part = "all") %>%
      hline(i = 26, border = fp_border()) %>%
          rotate(j = 1:dim(Tabla)[2], rotation = "btlr", part = "header") %>%
            height(i = 1, h_header-0.4, part = "header") %>%
              hrule(i = 1, rule = "exact", part = "header") %>%
                width(j = 1: ncol(Tabla), width = c(1.4, rep(0.4,
ncol(Tabla)-1))) %>%
                  fontsize(size = 8.5, part = "all") %>%
                    set_caption(caption = "Variability (standard deviation)
of the log(SMRs) for each disease in women and city.", autonum =
run_autonum(pre_label = "Table ", post_label = ". "))


Tabla
```

Table 2. Variability (standard deviation) of the log(SMRs) for each disease in women and city.

	Diabetes	Dementia	IHD	COPD	Ictus	Cirrhosis	Stomach c.	Lung c.	Breast c.	Colorectal c.	Haematological c.	Mean
Alicante	0.16	0.21	0.13	0.13	0.10	0.19	0.04	0.06	0.07	0.04	0.05	0.11
Almería	0.19	0.20	0.27	0.08	0.18	0.05	0.08	0.11	0.10	0.14	0.10	0.14

	Diabetes	Dementia	IHD	COPD	Ictus	Cirrhosis	Stomach c.	Lung c.	Breast c.	Colorectal c.	Haematological c.	Mean
Avilés	0.05	0.24	0.10	0.11	0.22	0.07	0.02	0.05	0.15	0.05	0.06	0.10
Barcelona	0.26	0.24	0.19	0.27	0.15	0.21	0.13	0.20	0.14	0.10	0.10	0.18
Bilbao	0.19	0.23	0.10	0.18	0.08	0.21	0.22	0.16	0.04	0.06	0.03	0.14
Cádiz	0.15	0.10	0.19	0.07	0.11	0.16	0.13	0.08	0.08	0.07	0.14	0.12
Cartagena	0.19	0.18	0.20	0.24	0.23	0.20	0.07	0.05	0.18	0.17	0.11	0.16
Castellón	0.07	0.15	0.10	0.06	0.09	0.05	0.04	0.10	0.02	0.07	0.04	0.07
Córdoba	0.17	0.20	0.21	0.18	0.17	0.12	0.20	0.09	0.10	0.09	0.12	0.15
Gijón	0.17	0.13	0.15	0.13	0.12	0.04	0.11	0.05	0.09	0.06	0.02	0.10
Granada	0.29	0.14	0.20	0.16	0.21	0.26	0.20	0.07	0.09	0.11	0.09	0.17
Huelva	0.26	0.27	0.20	0.18	0.20	0.14	0.07	0.12	0.10	0.12	0.08	0.16
Jaén	0.27	0.17	0.16	0.25	0.12	0.22	0.27	0.07	0.04	0.13	0.09	0.16
Madrid	0.21	0.24	0.15	0.22	0.13	0.12	0.11	0.20	0.08	0.08	0.05	0.14
Málaga	0.22	0.25	0.14	0.11	0.15	0.16	0.11	0.06	0.07	0.09	0.04	0.13
Murcia	0.33	0.24	0.17	0.17	0.19	0.14	0.12	0.11	0.09	0.10	0.11	0.16
Oviedo	0.24	0.10	0.11	0.11	0.09	0.09	0.16	0.06	0.04	0.04	0.04	0.10
Palma de Mallorca	0.34	0.40	0.25	0.23	0.21	0.10	0.15	0.13	0.17	0.12	0.08	0.20
Palmas Gran Canaria	0.31	0.10	0.19	0.22	0.18	0.14	0.18	0.07	0.08	0.09	0.14	0.16
Pamplona	0.21	0.25	0.19	0.21	0.16	0.08	0.05	0.05	0.06	0.12	0.05	0.13
San Sebastián	0.34	0.31	0.17	0.24	0.17	0.19	0.18	0.07	0.07	0.06	0.08	0.17
Santa Cruz Tenerife	0.20	0.08	0.13	0.09	0.09	0.17	0.02	0.10	0.09	0.05	0.05	0.10
Santander	0.19	0.22	0.17	0.17	0.19	0.09	0.05	0.05	0.10	0.08	0.02	0.12
Sevilla	0.27	0.20	0.22	0.20	0.22	0.31	0.17	0.12	0.12	0.10	0.08	0.18
Valencia	0.17	0.12	0.09	0.13	0.10	0.14	0.06	0.10	0.06	0.08	0.07	0.10
Vitoria	0.14	0.23	0.22	0.21	0.10	0.06	0.05	0.07	0.09	0.08	0.08	0.12
Mean	0.21	0.20	0.17	0.17	0.15	0.14	0.11	0.09	0.09	0.09	0.07	

```
# log(SMRs) standard deviations comparison as a function of cause of death
and city

# Women
sex <- 2
City <- rep(medea3_cities$codigo, dim(desv_log_SMRs[[sex]])[2])
Disease<- rep(colnames(desv_log_SMRs[[sex]]), each =
dim(desv_log_SMRs[[sex]])[1])
Variability <- c(desv_log_SMRs[[sex]])

data_fit <- data.frame(City, Disease, Variability)

fit_anova <- aov(Variability ~ Disease + City, data = data_fit)

print(summary(fit_anova))
```

3. Correlation plots

```
array_matcor_sp <- list()

array_matcor_sp$Men <- simplify2array(matcor_sp$hombres)
array_matcor_sp$Women <- simplify2array(matcor_sp$mujeres)

# Correlation matrix between causes of death (mean value for all the cities)
matrix_corr <- list()
matrix_corr$mean <- apply(array_matcor_sp$Men, c(1, 2), mean)
dimnames(matrix_corr$mean)[[1]] <- Causes$Men
dimnames(matrix_corr$mean)[[2]] <- Causes$Men

# Correlation 95% CIs
matrix_corr$lowCI <- apply(array_matcor_sp$Men, c(1, 2), function(x)
quantile(x, probs = 0.025))
matrix_corr$uppCI <- apply(array_matcor_sp$Men, c(1, 2), function(x)
quantile(x, probs = 0.975))
# Prob(correlation>0)
matrix_corr$Prob <- apply(array_matcor_sp$Men, c(1, 2), function(x) mean(x >
0))

corrplot(matrix_corr$mean, type = "upper" , method = "color", order = "FPC",
tl.pos = "tl", addCoef.col = "black", number.cex = 0.8, tl.col = "black",
tl.srt = 45, outline = TRUE)
corrplot_plotCI(matrix_corr$mean, add = TRUE, type = "lower", method =
"circle", order = "FPC",
         diag = FALSE, tl.pos = "n", cl.pos = "n", plotCI = "rect", lowCI.mat
= matrix_corr$lowCI, uppCI.mat = matrix_corr$uppCI, segments.col = "red",
segments.lty = 2, segments.lwd = 1)
```

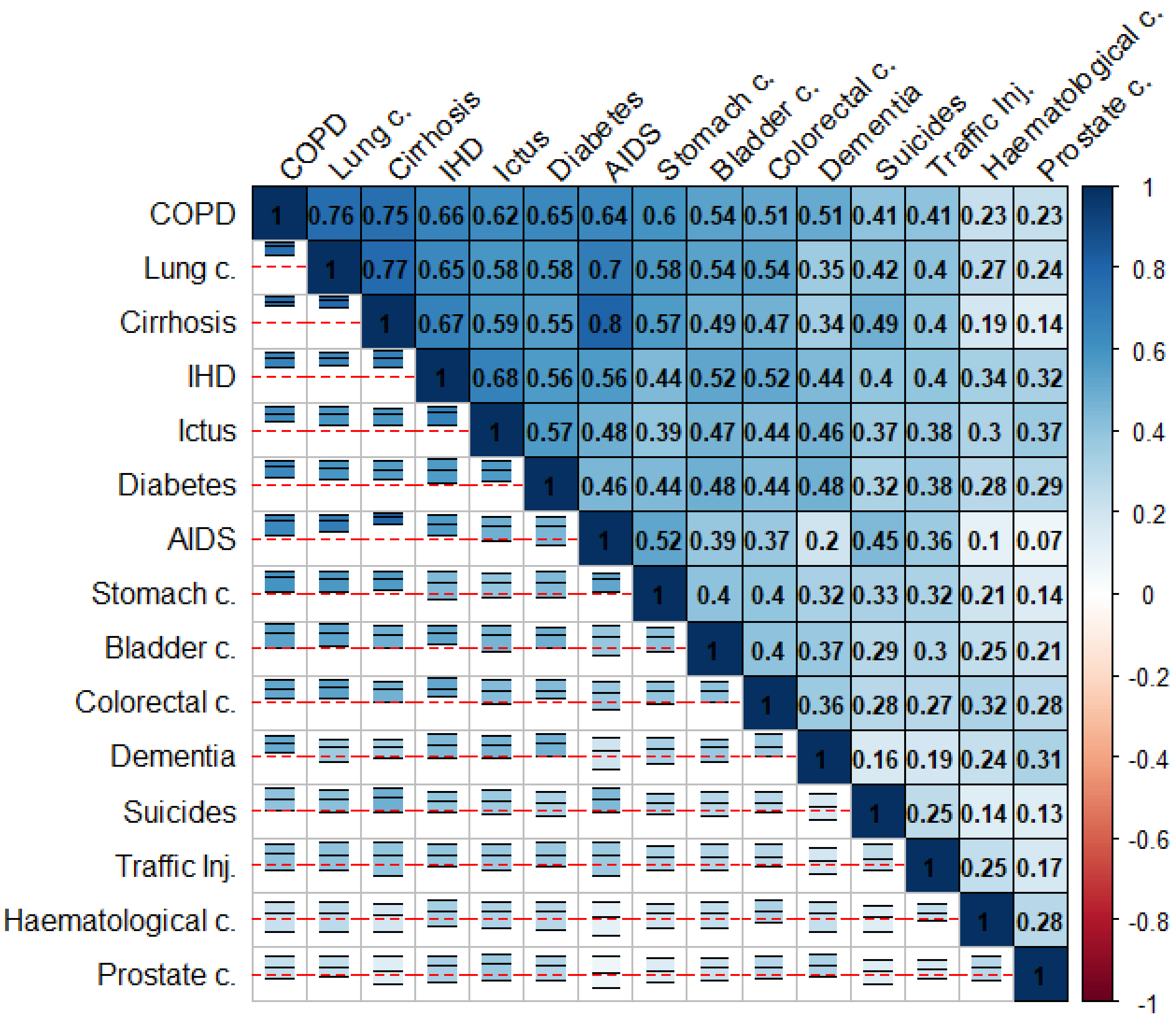

Figure 1: Mean correlation between diseases (upper diagonal) and CI (95%) (lower diagonal) in men.

```
# Women

# Correlation matrix between causes of death (mean value for all the cities)
matrix_corr <- list()
matrix_corr$mean <- apply(array_matcor_sp$Women, c(1, 2), mean)
dimnames(matrix_corr$mean)[[1]] <- Causes$Women
dimnames(matrix_corr$mean)[[2]] <- Causes$Women
```

```r
# Correlation 95% CIs
matrix_corr$lowCI <- apply(array_matcor_sp$Women, c(1, 2), function(x)
quantile(x, probs = 0.025))
matrix_corr$uppCI <- apply(array_matcor_sp$Women, c(1, 2), function(x)
quantile(x, probs = 0.975))
# Prob(correlation>0)
matrix_corr$Prob <- apply(array_matcor_sp$Women, c(1, 2), function(x) mean(x
> 0))

corrplot(matrix_corr$mean, type = "upper" , method = "color", order = "FPC",
tl.pos = "tl", addCoef.col = "black", number.cex = 0.8, tl.col = "black",
tl.srt = 45, outline = TRUE)
corrplot_plotCI(matrix_corr$mean, add = TRUE, type = "lower", method =
"circle", order = "FPC",
         diag = FALSE, tl.pos = "n", cl.pos = "n", plotCI = "rect", lowCI.mat
= matrix_corr$lowCI, uppCI.mat = matrix_corr$uppCI, segments.col = "red",
segments.lty = 2, segments.lwd = 1)
```

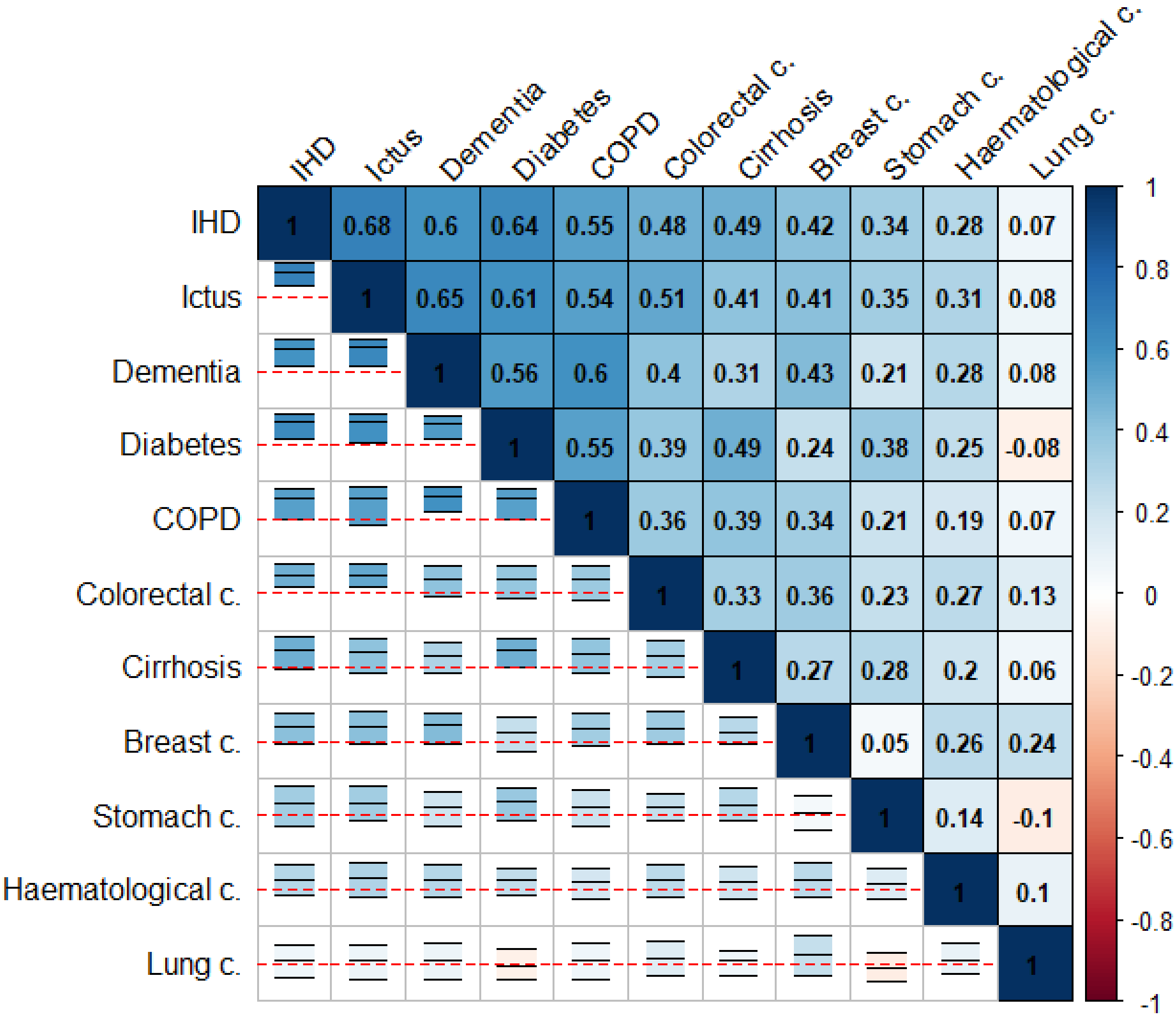

Figure 2: Mean correlation between diseases (upper diagonal) and CI (95%) (lower diagonal) in women.

4. Choropleth maps for Madrid and Barcelona

```
# Causes ID
dimnames(res_finales_sp$hombres)[[2]] <- Causes$Men

# SMRs for COPD, Stomach and Prostate Cancer in men for Madrid and Barcelona
```

```r
# Madrid and Barcelona
carto <- list()
carto$Madrid <- st_geometry(carto_medea3_sf[which(carto_medea3_sf$NMUN ==
"Madrid"),])
carto$Barcelona <- st_geometry(carto_medea3_sf[which(carto_medea3_sf$NMUN ==
"Barcelona"),])

# Cut points, palette and graphical parameters
cuts_SMR <- c(0, 0.70, 0.80, 0.90, 1.10, 1.20, 1.30)
palette <- brewer.pal(7, "BrBG")[7:1]
par(oma = c(0, 5, 0, 0), mar = c(0, 0, 1.5, 1), mfrow = c(3, 2))

for(j in c("COPD", "Stomach c.", "Prostate c.")){

  # Madrid

  id_secciones <- which( substr(rownames(res_finales_sp$hombres), 1, 5) %in%
medea3_cities$codigo[which(medea3_cities$nombre_municipio == "Madrid")] )

  aux <- findInterval(res_finales_sp$hombres[id_secciones, j, "RMEs"],
cuts_SMR)
  plot(carto$Madrid, col = palette[aux], xlim = c(-3.808947, -3.558235), ylim
= c(40.402064, 40.463334))

  if(j == "COPD"){ mtext("Madrid", side = 3, cex = 1.2, line = 0.2) }
  mtext(j, side = 2, cex = 1.2, line = 0.4)

  # Barcelona

  id_secciones <- which( substr(rownames(res_finales_sp$hombres), 1, 5) %in%
medea3_cities$codigo[which(medea3_cities$nombre_municipio == "Barcelona")] )

  aux <- findInterval(res_finales_sp$hombres[id_secciones, j, "RMEs"],
cuts_SMR)
  plot(carto$Barcelona, col = palette[aux], xlim = c(2.162453, 2.179207),
ylim = c(41.356925, 41.448253))

  if(j == "COPD"){ mtext("Barcelona", side = 3, cex = 1.2, line = 0.2) }

}
```

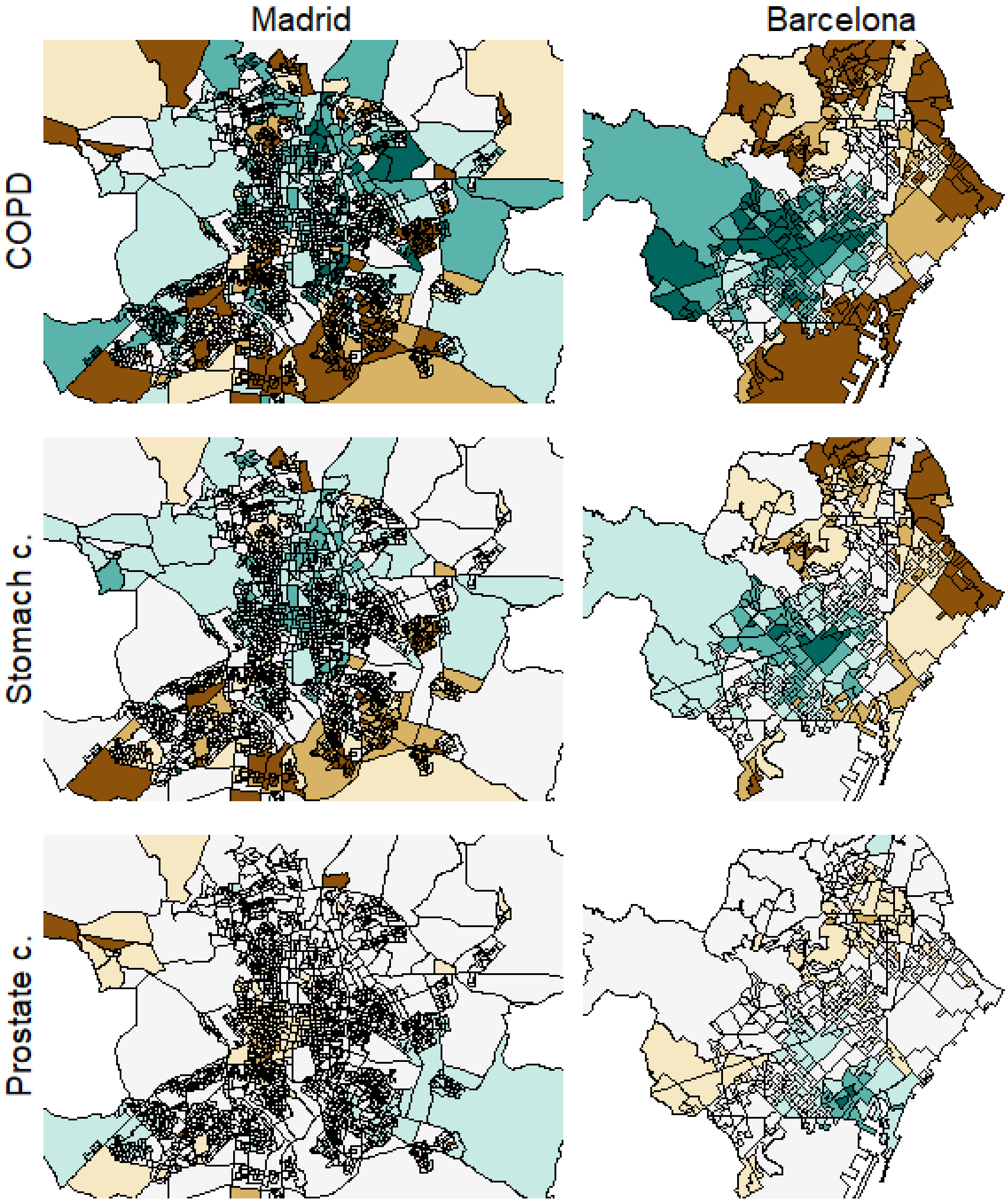

Figure 3: Choropleth maps for the estimated risk patterns for COPD, stomach cancer and prostate cancer in Madrid (left) and Barcelona (right).
