# Supplementary material for: Geographical Variability in Mortality in Urban Areas: A Joint Analysis of 16 Causes of Death
Source: Int J Environ Res Public Health. 2021 May 25;18(11):5664. doi: 10.3390/ijerph18115664 (PMC8197960; doi:10.3390/ijerph18115664)
Supplement: Supplementary file 1 [file ijerph-18-05664-s001.zip › Table S1.pdf]

Table S1. Causes of death considered for the MEDEA3 project.

Cause	Short term	ICD-9 codes	ICD-10 codes	Number of deaths, men	Number of deaths, women
AIDS	AIDS	279(.5.6)	B20-B24	9,466	2,381
Malignant neoplasm of stomach	Gastric cancer	151	C16	16,962	11,602
Malignant colorectal neoplasm	Colorectal cancer	153-154	C18-C21	41,407	32,826
Malignant neoplasm of trachea, bronchus and lung	Lung cancer	162	C33-C34	89,881	18,421
Malignant neoplasm of breast	Brest cancer	174	C50	235	35,393
Malignant neoplasm of prostate	Prostate cancer	185	C61	27,574	0
Malignant neoplasm of bladder	Bladder cancer	188	C67	19,775	4,893
Hematologic malignant neoplasm	Hematologic cancer	200-208, 273.3	C81-C96	21,469	20,245
Diabetes Mellitus	Diabetes mellitus	250	E10-E14	18,544	26,421
Organic senile and presenile mental disorders and Alzheimer's disease	Dementia	290, 331.0	F00-F09, G30	32,464	77,480
Ischemic heart diseases	Ischemic heart disease	410-414	I20-I25	108,892	88,797
Cerebrovascular diseases	Ictus	430-434, 436-438	I60-I69	65,047	98,221
Chronic obstructive pulmonary diseases	COPD	490-492, 494-496	J40-J44, J47	55,681	18,391
Cirrhosis and chronic liver diseases	Liver cirrhosis	571	K70, K72.1, K73-K74, K76.1.9	22,277	11,026
Suicide and intentional self-harm	Suicide	E950-E959	X60-X84	10,418	4,320
Motor vehicle accident injuries	Traffic accident	E810-E819	‡	9,998	3,472

‡ ICD10 codes: V02-V04(.1.9), V09(.2.3), V12-V14.(3.4.5.9), V19(.4.5.6.9), V20-V28(.3.4.5.9), V29(.4.5.6.9), V20-V28(.3.4.5.7.9), V29(.4.5.6.9), V30-V38(.3.4.5.7.9), V39(.4.5.6.9), V40-V48(.3.4.5.7.9), V49(.4.5.6.9), V50-V58(.3.4.5.7.9), V59(.4.5.6.9), V60-V68(.3.4.5.7.9), V69(.4.5.6.9), V70-V78(.3.4.5.7.9), V79(.4.5.6.9), V80(.3.4.5), V81-V82(.1), V83-V86(.0.1.2.3), V87(.0.1.2.3.4.5.6.7.8), V89(.2.9).
